# Evaluation of Magnesium-Phosphate Particle Incorporation into Co-Electrospun Chitosan-Elastin Membranes for Skin Wound Healing

**DOI:** 10.3390/md20100615

**Published:** 2022-09-29

**Authors:** Alex Bryan, Ethan Wales, Samarth Vedante, Andreu Blanquer, Dipesh Neupane, Sanjay Mishra, Lucie Bačáková, Tomoko Fujiwara, Jessica Amber Jennings, Joel D. Bumgardner

**Affiliations:** 1 Department of Biomedical Engineering, UofM-UTHSC Joint Graduate Program in BME, The University of Memphis, Memphis, TN 38152, USA; 2 Laboratory of Biomaterials and Tissue Engineering, Institute of Physiology of the Czech Academy of Sciences, Videnska 1083, 14220 Prague, Czech Republic; 3 Department of Physics, The University of Memphis, Memphis, TN 38152, USA; 4 Department of Chemistry, The University of Memphis, Memphis, TN 38152, USA

**Keywords:** electrospinning, chitosan, elastin, tissue engineering, wound healing

## Abstract

Major challenges facing clinicians treating burn wounds are the lack of integration of treatment to wound, inadequate mechanical properties of treatments, and high infection rates which ultimately lead to poor wound resolution. Electrospun chitosan membranes (ESCM) are gaining popularity for use in tissue engineering applications due to their drug loading ability, biocompatibility, biomimetic fibrous structure, and antimicrobial characteristics. This work aims to modify ESCMs for improved performance in burn wound applications by incorporating elastin and magnesium-phosphate particles (MgP) to improve mechanical and bioactive properties. The following ESCMs were made to evaluate the individual components’ effects; (C: chitosan, CE: chitosan-elastin, CMg: chitosan-MgP, and CEMg: chitosan-elastin-MgP). Membrane properties analyzed were fiber size and structure, hydrophilic properties, elastin incorporation, MgP incorporation and in vitro release, mechanical properties, degradation profiles, and in vitro cytocompatibility with NIH3T3 fibroblasts. The addition of both elastin and MgP increased the average fiber diameter of CE (~400 nm), CMg (~360 nm), and CEMg (565 nm) compared to C (255 nm). Water contact angle analysis showed elastin incorporated membranes (CE and CEMg) had increased hydrophilicity (~50°) compared to the other groups (C and CMg, ~110°). The results from the degradation study showed mass retention of ~50% for C and CMg groups, compared to ~ 30% seen in CE and CEMg after 4 weeks in a lysozyme/PBS solution. CMg and CEMg exhibited burst-release behavior of ~6 µg/ml or 0.25 mM magnesium within 72 h. In vitro analysis with NIH3T3 fibroblasts showed CE and CEMg groups had superior cytocompatibility compared to C and CMg. This work has demonstrated the successful incorporation of elastin and MgP into ESCMs and allows for future studies on burn wound applications.

## 1. Introduction

The USA spends ~$20B per year on wound treatments, including severe burn injuries requiring emergency room treatment and hospitalization [1]. Nearly 400,000 patients experienced severe burns that needed emergency treatment in 2018 [2]. Though most patients survive, those with severe damage may have permanent loss of function or scarring. In addition, the disability and disfigurement that accompanies severe burns (2nd/3rd degree) affect patients’ daily life tasks and contribute to social and economic hardships [3,4].

Standard treatments for severe burns use auto-, allo-, or xeno- grafts [5,6]. These treatments rely on explanted skin tissue covering damaged tissue and acting as a platform for new tissue growth. Skin grafts provide the damaged environment with protection, a structural template, and biological cues for healing and regeneration [6]. However, these treatments are still exposed to the external environment and are prone to infection [7]. Grafting results in inadequate aesthetics due to hyperpigmentation and disfigurement, impacting the patient’s social and economic well-being [1,4]. Autografts may not be an option for some patients as severe burns often cover a significant portion of skin, leaving little to explant. Allografts and xenografts avoid the issue of harvesting tissue from the patient, but because they are derived from other individuals or animal sources, they may induce unwanted inflammation caused by an immune response [8,9]. 

There has been much research to overcome these challenges by developing tissue engineered burn treatment templates to mimic the extracellular matrix (ECM) of skin and deliver pro-healing biological agents and/or healthy cells to stimulate tissue healing, growth, and regeneration [5,10]. Treatments are categorized by the layer(s) of skin that are meant to be mimicked/healed (epidermal, dermal, or both) with severe injuries requiring healing of both layers [11]. Epidermal treatments include delivering keratinocytes to the injury through sheets, sprays, or dressings. Dermal treatments focus on either providing a natural/synthetic dermis matrix/dressing to stimulate fibroblast growth, deliver donor fibroblasts, or act as an epidermal barrier [11]. Least in abundance are full thickness treatments which require either autologous or allogeneic keratinocytes and fibroblasts delivered on a temporary skin-mimicking template [11].

Though these tissue engineered cell delivery treatments show promise, their use is limited due to poor mechanical properties, lack of tissue-template integration, inadequate vascularization, high cost, and are labor intensive [8,11,12]. Epicel, an autologous epithelial treatment, requires a 2–3 week period to culture keratinocytes before patient application [13]. Furthermore, this treatment can cost $6000–$10,000 per 1% body surface area limiting its access to rural areas and low income patients [13]. Other currently available treatments, like Dermagraft and Apligraf, involve multiple steps to harvest and culture allogenic cells resulting in similar production costs and waiting times [13]. The performance of these tissue engineered treatments are highly technique sensitive; they are often mishandled during application and require increased cleaning and attention from caregivers [5,14,15]. Additionally, more than 90% of burn-associated deaths occur in low and middle-income countries which suggests the need for a less costly and technology dependent treatment [16].

Addressing these drawbacks may improve overall healing outcomes and accessibility of the treatment. The biopolymer, chitosan, has gained considerable attention for use as a skin wound treatment material due to its many pro-healing characteristics [17,18]. Chitosan is a readily available biomaterial derived from the exoskeleton of crustaceans that has shown promise for tissue engineering applications [12,19]. Some of its natural properties, like clot promotion, anti-inflammatory, and antimicrobial properties, are advantageous for burn wound applications [17]. Alongside these properties, chitosan can act as a drug delivery vehicle providing an additional opportunity to influence healing [18]. Chitosan treatments are usually delivered in a hydrogel or dressing accompanied by other bioactive polymers or agents [17,18,20]. Recently, there has been progress made in utilizing electrospun chitosan for wound healing applications which include combinations with synthetic polymers like poly(vinyl-alcohol), polyethylene oxide, and polycaprolactone [21,22,23,24]. Some of these materials also have successfully delivered bioactive components like silver nanoparticles and zinc oxide to elevate wound healing performance in vivo. Though various combinations of chitosan-copolymer and chitosan-drug burn treatments have been explored [17,20,25,26], there are many therapeutic combinations which may further improve healing that have yet to be tested. 

Our past studies have demonstrated electrospun chitosan membranes (ESCM) are successful in tissue engineering applications for bone regeneration and burn wound treatment [27,28,29]. Because of their biomimetic fibrous structure and potential for drug delivery, these membranes may be further modified to help enhance their bioactivity to support skin healing and re-epithelialization that would lead to improved burn wound healing performance. 

Elastin was selected to be incorporated into ESCMs because it is the second-most abundant matrix component found in the body, its elastic mechanical properties, and its ability to support cell adhesion and growth [30,31]. The elastin-chitosan composite structure may provide amino acid sequences which serve as ligands for adhesion receptors on cells which are lacking in chitosan’s polysaccharide structure. The inclusion of elastin may also improve the mechanical properties to better facilitate healing of skin, compared to chitosan alone [18,31]. 

Magnesium-phosphate particles (MgP) were selected to be incorporated into the ECM to deliver magnesium because it is a commonly found ion in the body, plays a role across multiple systems, and may improve wound healing outcomes [32,33]. Magnesium has been shown to improve regeneration in full thickness skin wounds and may be attributed to the promotion of vascularization [34,35,36]. A protocol established by Zhou et al. [37] was selected as the method for MgP production due to our previous success using a similar synthesis method for calcium phosphate-silver nanoparticles incorporation into chitosan coatings [38].

Though we have previously been successful in incorporating elastin into ESCMs, the novelty of this work is the assessment of the feasibility of loading MgP into the ESCMs in addition to elastin. This work aims to evaluate the physical, mechanical, degradation, and cytocompatibility properties of electrospun chitosan-elastin co-electrospun membranes loaded with MgP for skin wound healing applications. It is believed that incorporating elastin and MgP into ESCM may improve their physical, chemical, and bioactive properties for improved healing performance in burn wounds. Electrospun membranes were characterized for physical structure and chemical composition and in vitro degradation, magnesium release, and cytocompatibility.

## 2. Results

### 2.1. Characterization

#### 2.1.1. Fiber Morphology and Fiber Diameter Analysis

**Figure 1:**
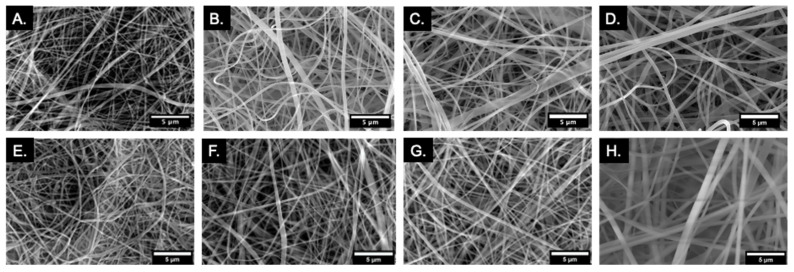
SEM images display uniform fiber morphology of pre-treatment (**A**–**D**) and post-treatment (**E**–**H**) C (Chitosan), CE (Chitosan-Elastin), CMg (Chitosan-MgP), and CEMg (Chitosan-Elastin-MgP) membranes. Images taken on NOVA NanoSEM 650 (FEI Company, Hillsboro, OR, USA) at 5000 ×.

SEM imaging showed uniform and smooth fiber morphology across all membrane groups (Figure 1). There were small amounts of fiber fusion but no evidence of fiber beading. In addition, there was little difference in fiber morphology of membranes before (Figure 1A–D) and after (Figure 1E–H) tBOC treatment. C membranes had an average fiber diameter of 255 nm (Figure 1A,C). After the inclusion of elastin alone, CE membranes’ fiber diameter increased to 400 nm (Figure 1B,F). Similarly, CMg membranes saw an increase to 360 nm (Figure 1C,G) with the inclusion of MgP alone. When both elastin and MgP were added, CEMg fiber diameter further increased to 565 nm (Figure 1D,H). 

**Figure 2:**
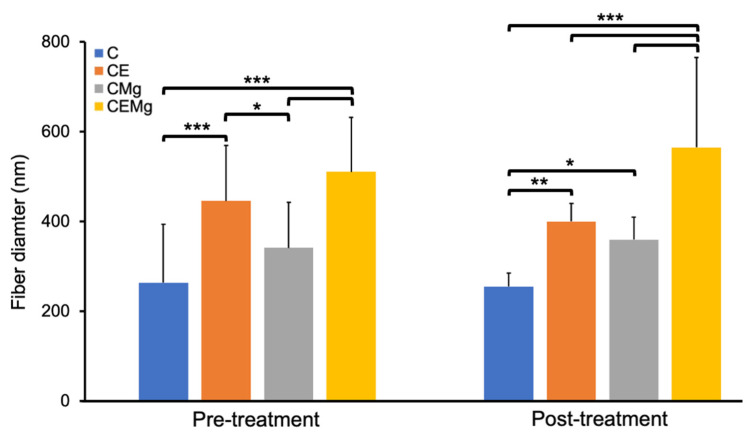
Bar graph of average fiber diameter trend for CE, CMg, and CEMg membranes before and after tBOC treatment (*n* = 3). * denotes significant difference (*p* < 0.05). ** denotes significant difference (*p* < 0.001). *** denotes significant difference (*p* < 1 × 10^−5^).

Statistical analysis of fiber diameter found that post-treatment CE (*p* = 0.0005), CMg (*p* = 0.021), and CEMg (*p* = 4 × 10^−14^) membranes had significantly larger fiber diameters than the control chitosan (C) membranes (Figure 2). Additionally, the CEMg membrane’s fiber diameter was statistically different from CE (*p* = 1 × 10^−6^) and CMg (*p* = 8 × 10^−9^). Further analysis of pre- and post-treatment versions of membranes confirmed no significant differences for all groups (*p* = 0.86, *p* = 0.22, *p* = 0.31, *p* = 0.053; for C, CE, CMg, and CEMg respectively. 

#### 2.1.2. EDS Analysis for MgP Incorporation Verification 

**Figure 3:**
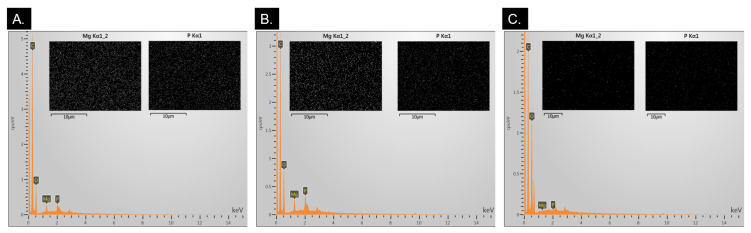
EDS spectra analysis of post-treated CMg (**A**), CEMg (**B**), and C (negative control) (**C**) confirm Mg and P incorporation into membranes via magnesium peak (~1.3 keV) and phosphorus peak (~2 keV). Images showing visual mapping of magnesium and phosphorus are included for each respective group and displays uniform distribution. * Note: Images have had saturation and sharpness increased and converted to black and white to improve visualization.

The EDS spectra showed strong peaks at ~1.25 keV and at ~2 keV which corresponds to the Kα peaks for magnesium and phosphorus, respectively, in CMg and CEMg membranes (Figure 3). The presence of these peaks confirmed the successful incorporation of magnesium into the electrospun. EDS visual mapping of the magnesium and phosphorus peaks showed a uniform distribution of magnesium and phosphorus as white dots. These data indicate that the addition of the MgP particles to the spinning solution lead to their incorporation and retention in the membranes during the electrospinning fabrication and post-tBOC treatment processes. 

#### 2.1.3. FTIR Analysis

**Figure 4:**
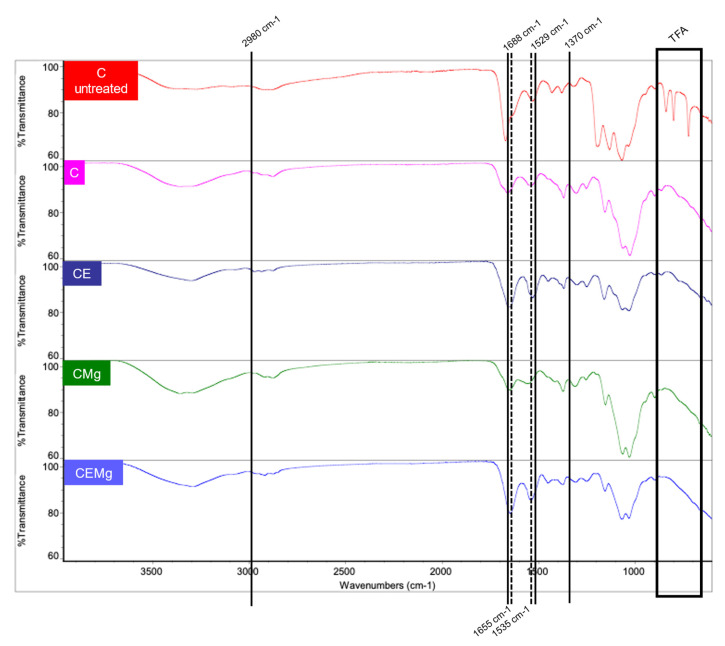
FTIR spectra of as spun C (untreated) membranes shows peaks at 720, 802, and 837 cm^−1^ (black box in graph) indicating presence of residual TFA salts due to spinning process. These peaks are missing in FTIR spectra of the C, CE, CMg and CEMg membranes after post-spinning -tBOC treatment indicating removal of the TFA salts from the membranes. tBOC treatment. tBOC treatment-related peaks are individually labeled with black lines (1370, 1529, 1688, and 2980 cm^−1^). Elastin-related peaks are marked with a dashed line (1535 and 1655 cm^−1^).

FTIR analysis of untreated C and post-treatment C, CE, CMg, and CEMg membranes is shown in Figure 4. All post-treatment membrane groups lacked peaks at 720, 802, and 837 cm^−1^ characteristic of trifluoroacetic acid (TFA) salts, confirming successful salt removal. The elastin-incorporated membranes (CE and CEMg) exhibited FTIR peaks at wavenumbers associated with the elastin amide (1535 and 1655 cm^−1^) bonds. The presence of these peaks confirms the presence of the elastin in the electrospun membranes. 

#### 2.1.4. X-Ray Diffraction (XRD) Crystallography Analysis

**Figure 5:**
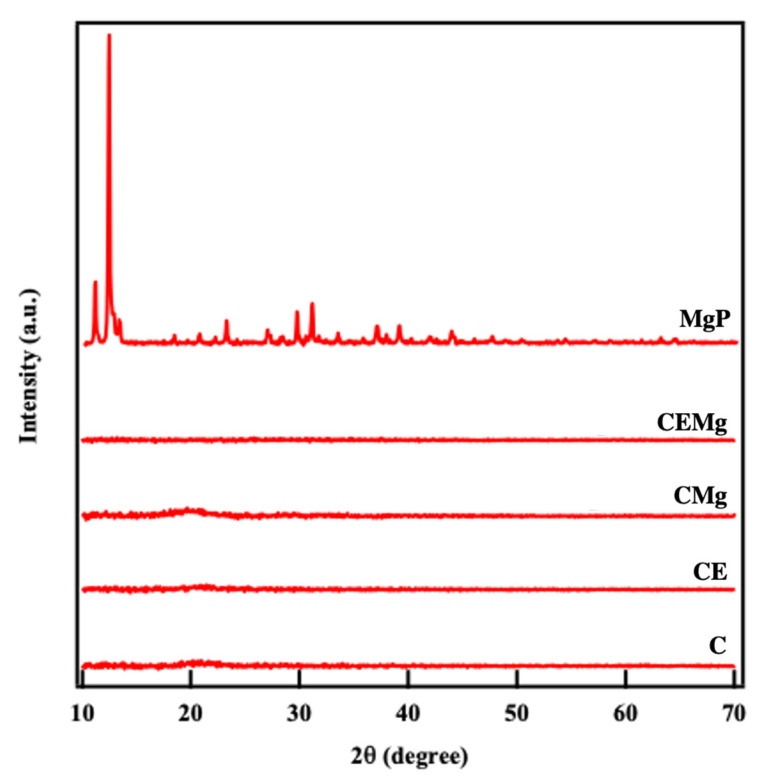
XRD results show strong peak for MgP indicating they are in a highly crystalline structure. Lack of peaks in electrospun C, CE, CMg and CEMg membranes indicate loss of crystallinity of MgP and all membranes had an amorphous chitosan structure.

XRD analysis of MgP alone and post-treatment membranes are shown in Figure 5. Spectra for the as formed MgP showed sharp peaks corresponding to crystalline magnesium orthophosphate [Mg_3_(PO_4_)_2_-8H_2_O] [37]. There are no peaks seen in the XRD spectra for the CMg and CEMg membrane groups, indicating loss of MgP particle crystallinity. Additionally there are no peaks observed for chitosan in any of the electrospun membranes indicating amorphous structure.

#### 2.1.5. Water Contact Angle Analysis

**Figure 6:**
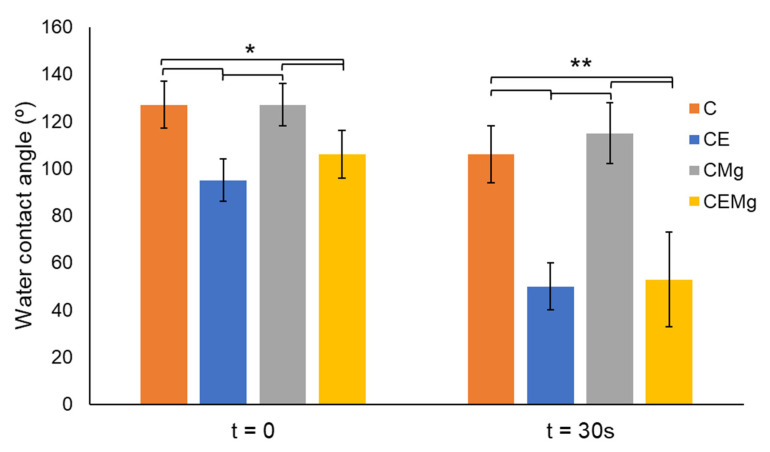
Water contact angle measurements (*n* = 3/group) show decreased values in elastin-incorporated membranes after 30 s of exposure to water droplet. *denotes significant difference (*p* < 0.01), ** denotes significant difference (*p* < 0.001).

Results of the water contact angle measurements are shown in Figure 6. Results indicated that all membrane groups were initially hydrophobic with contact angles greater than 90°. After 30 s, the water contact angles of the elastin-containing membranes decreased to angles much less than 90° as compared to the non-elastin-containing membranes. Statistical analysis showed that CE was significantly different than C (*p* = 0.003, *p* = 3 × 10^−15^) and CMg membranes (*p* = 0.0016, *p* = 7 × 10^−8^) at t = 0 s and at t = 30 s, respectively. Similarly, CEMg showed significant differences compared to C (*p =* 0.0094, *p =* 2 × 10^−5^) and CMg (*p =* 0.005, *p =* 2 × 10^−6^) at t = 0 s and t = 30 s, respectively.

#### 2.1.6. Immunofluorescence Staining for Elastin Incorporation

**Figure 7:**
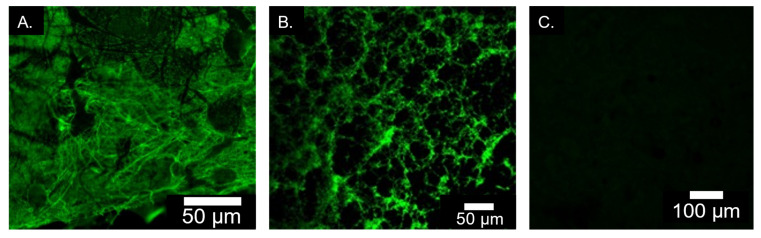
Representative immunofluorescence images confirming presence of elastin in CE (**A**), CEMg (**B**), and ((**C**), negative control). Green color indicates presence of elastin.

Results of the immunofluorescence staining for elastin in the membranes are shown in Figure 6. Images of the immunostained CE (Figure 7A) and CEMg (Figure 7B) membranes showed extensive green fluorescence indicating the successful incorporation into the ESCMs. Green fluorescence was not observed in the C or CMg membranes. The C-membrane (Figure 7C) is shown as representative of the non-elastin containing membranes. 

#### 2.1.7. MgP Size and Zeta Potential Analysis

**Figure 8:**
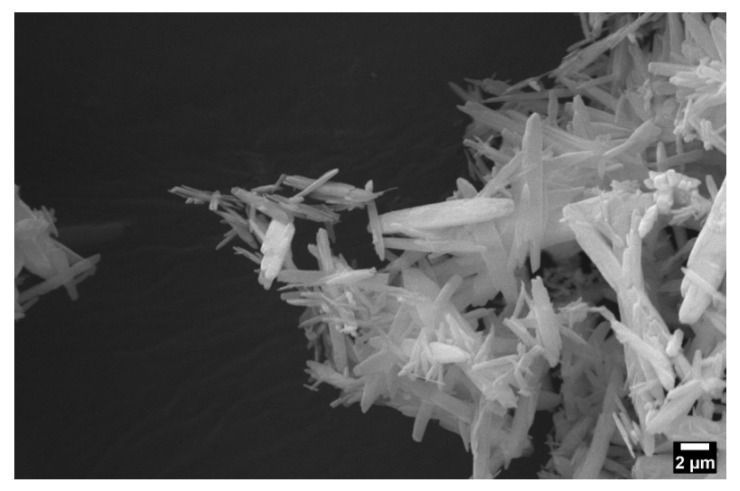
SEM images of MgP showed rod-like morphology. Images taken on NOVA NanoSEM 650 (FEI Company, Hillsboro, OR, USA).

SEM images of the MgP show an oblong crystalline morphology (Figure 8). Zeta potential and particle size are shown in Table 1. Zeta potential and particle size were calculated from three independent samples and averaged. MgP had an average zeta potential of -11.8 mV and an average particle size of 1660 nm. 

#### 2.1.8. Combustion Analysis

Ash content combustion analysis of the C, CE, CMg, and CEMg membranes is shown in Table 2. Membranes without MgP incorporation (C and CE) had lower ash content (~2–4%) compared to MgP incorporated membranes (7–9%) (CMg and CEMg).

#### 2.1.9. Tensile Testing

**Figure 9:**
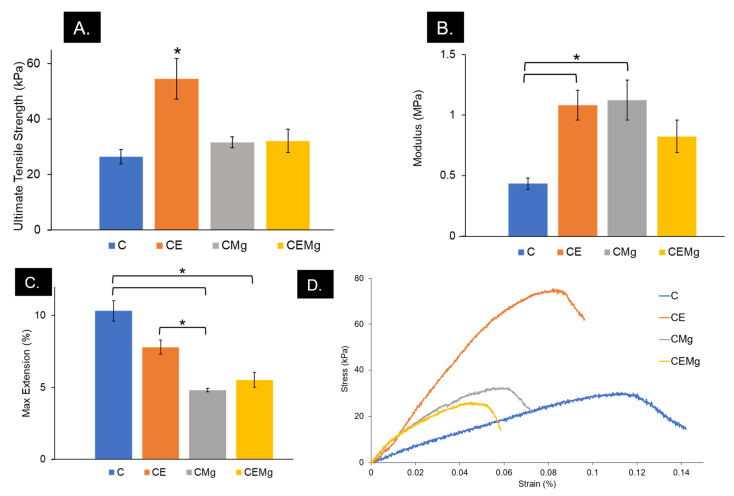
CE membranes had the highest ultimate tensile strength (kPa) (**A**). CE and CMg saw increased Young’s moduli (MPa) (**B**). The addition of elastin, MgP, or both decreased maximum extension (**C**) of membranes. * denotes significant difference (*p* < 0.05). Representative stress–strain curves of all membranes (**D**).

The result of mechanical tensile testing of the membranes is shown in Figure 9. One-factor ANOVA was used to identify differences in UTS, elastic modulus, and percent elongation among membrane types. The CE membranes had significantly greater UTS (~50 kPa) as compared to C (*p* = 3 × 10^−4^), CMg (*p* = 0.0012), and CEMg (*p* = 0.0014) (Figure 9A). The inclusion of elastin (CE, *p* = 0.05) and magnesium (CMg, *p* = 0.039) alone caused significant differences to be found in moduli when compared to C membranes, but not when incorporated together (CEMg, *p* = 0.29) (Figure 9B). Max extension was significantly impacted by the inclusion of elastin and MgP (Figure 9C). CMg (*p* = 7 × 10^−4^) and CEMg (*p* = 0.002) had significantly decreased extension compared to that of C. Additionally, CE max extension was also found to be different than CMg (*p* = 0.029).

### 2.2. In Vitro Analysis

#### 2.2.1. In Vitro Magnesium Release

**Figure 10:**
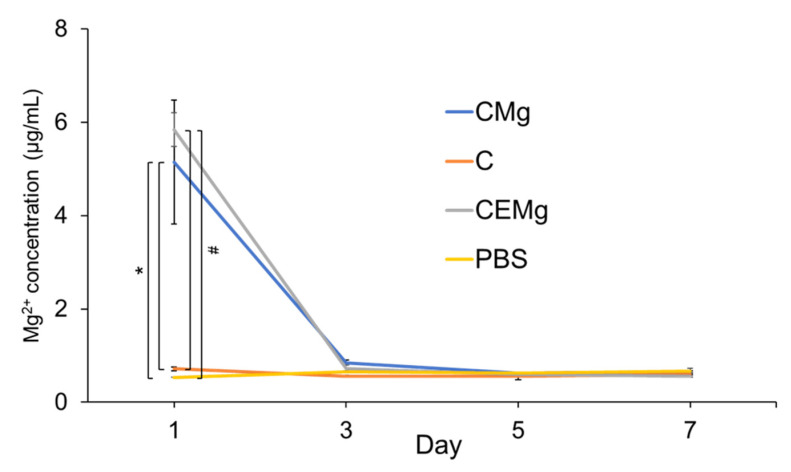
Magnesium release (*n* = 3/group) peaked on day 1 for CMg and CEMg groups with minimal release on subsequent timepoints and negative controls (C and PBS). * denotes significant difference (*p* < 0.05). # denotes significant difference (*p* < 0.01).

The 7-day magnesium release study results are shown in Figure 10. Each 1-cm disc membrane released approximately 6 µg/mL or 0.25 mM magnesium within 24 h. A two-factor ANOVA identified a significant interaction among the test variables (sample groups and time) on magnesium release (*p* = 4 × 10^−8^). Analysis also identified the significant differences between sample groups (*p* = 9 × 10^−6^) and over time (*p* = 1.9 × 10^−10^) on magnesium release. A one-factor ANOVA with Tukey HSD posthoc analysis for Day 1 showed CMg membranes were significantly different than C and PBS groups (*p* = 0.0184, *p* = 0.0149, respectively). The CEMg group’s magnesium release was also significantly different compared to C and PBS (*p* = 0.0082, *p* = 0.0067, respectively). No magnesium was released on days 3, 5, or 7.

#### 2.2.2. In Vitro Degradation Profiles of Membranes

**Figure 11:**
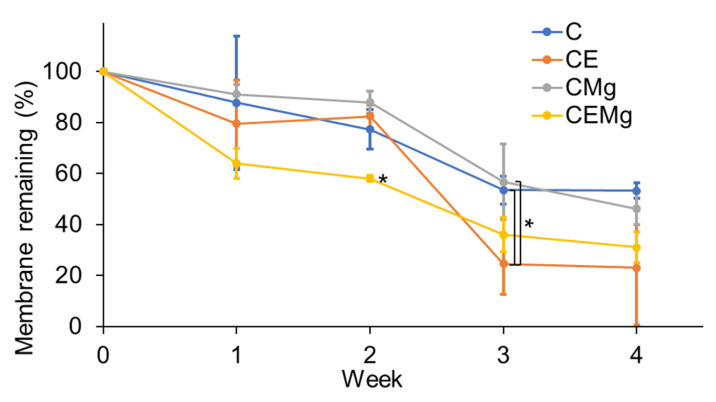
Degradation profiles of membrane groups showed increased rates for elastin-incorporated membranes by week 3. Membrane % remaining is a comparison of current mass vs. undegraded membrane mass. * denotes significant difference (*p* < 0.05).

Results of the in vitro degradation study are shown in Figure 11. All membranes showed significant decreases in mass over the 4-week test period. Over the initial two weeks, the elastin-containing membranes tended to show more mass loss than non-elastic membranes, especially after the first 2-weeks. By week 4, The CE and CEMg membranes showed approximately 70% loss in mass compared to only 50% loss of the C and CMg membranes. 

Statistical analysis using a two-factor ANOVA showed significant differences in membrane degradation between groups (*p* = 3 × 10^−6^), over time (*p* = 1 × 10^−16^), and that there was a significant interaction between membrane groups and time (*p =* 0.028). One-factor ANOVA within each timepoint was conducted and found that at week 2, CEMg was significantly different compared to C (*p* = 0.007), CE (*p* = 0.0014), and CMg (*p* = 0.00037) sample groups. Furthermore, at week 3, CE was found to be different compared to C (*p* = 0.039) and CMg (*p* = 0.023). No differences were found at weeks 1 and 4. 

#### 2.2.3. In Vitro MgP Cytotoxicity

**Figure 12:**
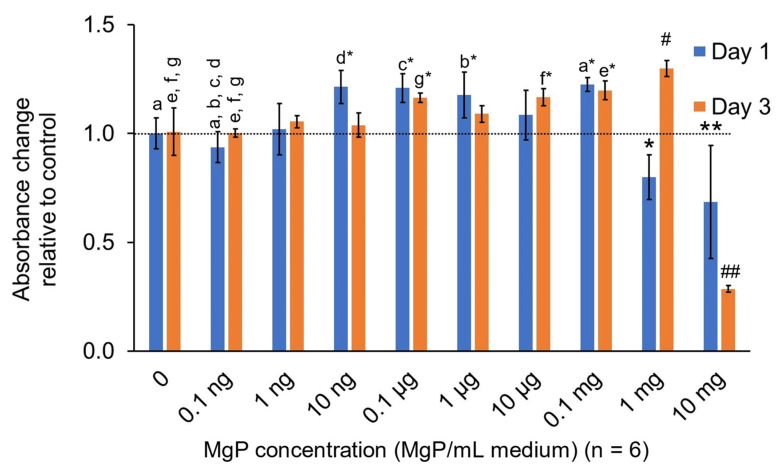
Absorbance from cytotoxicity assay after cell exposure to MgP-doped media for 24 and 72 h showed cytotoxicity beginning near 1 mg/mL–10 mg/mL (MgP/mL media). Data is normalized to media blank with no MgP (0 MgP/mL medium). Higher absorbance is associated with a higher cell count. a*–g* indicates a significant difference (*p* < 0.05) to all groups with matching letters (a–g). * indicates a significant difference (*p* < 0.01) to all groups except 0, 0.1 ng, and 1 ng MgP/mL medium within day 1. # indicates a significant difference (*p* < 0.001) to all groups except 0.1 mg, 10 μg, 0.1 μg MgP/mL medium within day 3. ** indicates a significant difference (*p* < 0.01) to all groups except 1 mg MgP/mL medium within day 1. ## indicates a significant difference (*p* < 1 × 10^−10^) to all groups within day 3.

MgP cytotoxicity results are shown in Figure 12. On Day 1, the viability of the cells exposed to up to 1 mg/ml MgP’s was not significantly reduced compared to the positive control (0 MgP group). The cells exposed to 10 mg/mL MgP exhibited a significant reduction in viability compared to the contro1 (*p* = 0.0004). Additionally, the only group to significantly increase cell viability on day 1 was 0.1 mg/mL MgP (*p* = 0.034). After 3 days, the 10 mg/mL group was still significantly reduced compared to control (*p* = 9 × 10^−15^). On day 3 the 0.1 µg/mL (*p* = 0.0254), 10 µg/mL (*p* = 0.0295), 0.1 mg/mL (*p* = 0.0027), and 1 mg/mL (*p* = 2 × 10^−6^) MgP groups all saw significant increases in cell viability compared to the positive control. The 1 mg/mL group had the largest increase in cytocompatibility from day 1 to 3.

#### 2.2.4. In Vitro Cytocompatibility of Membranes with NIH3T3 Fibroblasts

**Figure 13:**
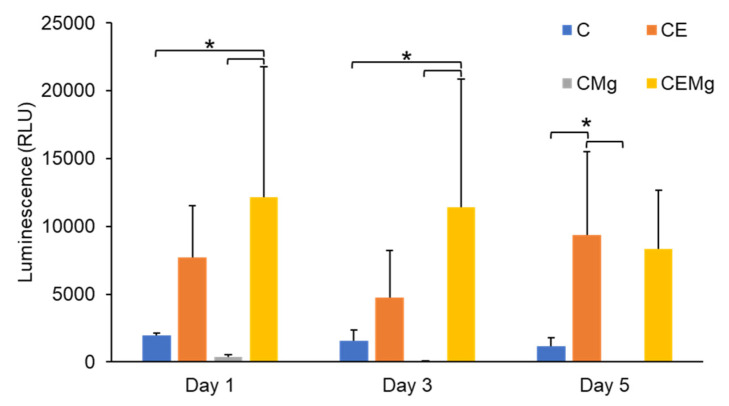
Luminescence of eluants collected at days 1, 3, and 5 from NIH3T3 fibroblasts on membranes showed increased cytocompatibility in CE and CEMg membranes. Luminescence is indicative of increased cell count. * denotes significant difference (*p* < 0.05).

Results of the cytocompatibility of the different ESCM groups with NIH3T3 fibroblasts are shown in Figure 13. A two-factor ANOVA did not find any significant interaction between test variables (membrane type and time) on cytocompatibility (*p* = 0.53). Further analysis indicated a significant difference in cytocompatibility among membrane groups (*p* = 2 × 10^−6^) but not over time (*p* = 0.5). One-factor ANOVA within each timepoint identified differences in cytocompatibility among groups. On day 1, CE and CEMg membranes showed greater cytocompatibility than other groups, but only CEMg groups were deemed statistically different from C (*p* = 0.032) and CMg (*p* = 0.012). On day 3, this trend continued with CEMg again being statistically different from C (*p* = 0.048) and CMg (*p* = 0.015). However, on day 5, CE proliferation had a large increase and was deemed statistically different compared to C (*p* = 0.042) and CMg (*p* = 0.025).

**Figure 14:**
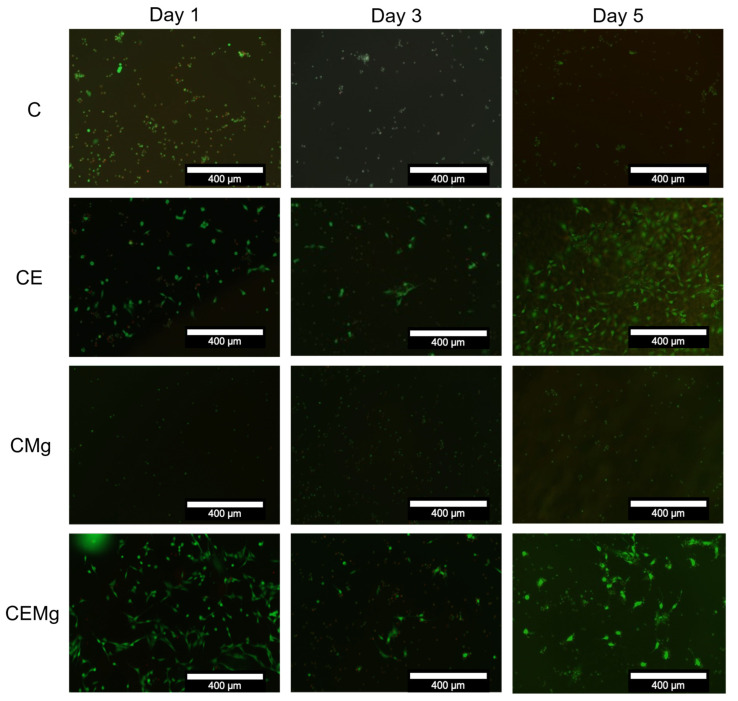
Images of C, CE, CMg, CEMg membranes at 1, 3, and 5 days cultured with NIH3T3 fibroblasts confirmed increased cytocompatibility with elastin-incorporated membranes. Images were made by overlaying ‘live’ and ‘dead’ stained images of the same location on the membrane.

Viability staining images, shown in Figure 14 correlate with the findings in the luminescence assay. Both C and CMg membranes showed low numbers of viable stained (green fluorescence) cells on day 1 with minimal increase in viable staining cells over the 5 days of culture. Viable cells that remained on these membranes exhibited spherical morphologies and did not spread. For the CE and CEMg membranes, there were greater numbers of viable stained cells on the membranes at day 1 and there appeared to be some increase in numbers of viable staining cells over the 5 days of culture. It was notable that the cells on the membranes incorporating elastin exhibited a more typical elongated and spread morphology as compared to the non-elastin incorporated membranes. 

## 3. Discussion

This work aimed to examine the potential of incorporating elastin and MgP particles to modify ESCMs for improved performance in burn wound applications. The addition of the elastin and MgP was intended to enhance the compatibility and mechanical properties of the membranes leading to overall improved wound healing outcomes. This initial work confirmed the increased bioactivity and mechanical properties due to elastin incorporation.

Incorporation of elastin and MgP into post-tBOC treated ESCMs was found to be successful via immunofluorescence staining and imaging of elastin and EDS, combustion, and in vitro release assessment for MgP. EDS analysis of CMg and CEMg both show peaks for magnesium and phosphorus suggesting incorporation of MgP. Combustion analyses also showed an increase in ash content of ~5% which corresponds to the mass of MgP particles added to the spinning solutions based on theoretical calculations XRD data showed that the MgP particles initially had a highly crystalline structure, which was not observed after manufacturing into electrospun membranes. The lack of MgP peaks in XRD spectra does not indicate a lack of MgP within the CMg and CEMg membranes, rather only indicates that the MgP particles are not in a crystalline state. It is likely that the crystalline MgP particles lost crystalline structure and dissolved in the acidic TFA/DCM solution used to electrospin the membranes. These data thus indicate that while not present in a crystalline state, that magnesium and phosphorus were incorporated in the CMg and CEMg membranes due to the addition of the MgP particles to the electrospinning solution.

Fiber diameter sizes increased with the addition of elastin and MgP. The increase in fiber size was attributed to an increase in viscosity of the spinning solution that was observed during solution preparations. The effect of increasing solution viscosity leading to increased electrospun fiber diameter aligns with prior reports [39,40]. The mechanism for this phenomenon is that, as the viscosity of the electrospinning solution increases, the forces of the molecules within the solution that resist stretching will increase. In turn, with no compensation of other parameters, this will cause an increase in fiber diameter [41].

Though the addition of elastin into the chitosan solution had less of an effect than MgP on fiber diameter, when both elastin and MgP are added simultaneously, the solution viscosity further increased, and subsequently fiber diameter also increased. Furthermore, because chitosan is an electroactive biopolymer, the incorporation of MgP may have contributed to increased fiber diameter due to ionic interactions during solving/electrospinning [42,43]. The zeta potential analysis showed an average charge of −11.8 mV for the MgP which further suggests ionic interactions with chitosan’s cationic amine group may have occurred. This increase in fiber diameter, however, is not a concern since fiber diameters are similar to other chitosan studies that have shown positive results for similar applications [44,45,46].

Water contact angle results indicated that all membrane groups were initially hydrophobic with contact angles greater than 90°. The hydrophobic characteristics is a result of the di-tert-butyl dicarbonate (tBOC) treatment which adds di-tert butyl dicarbonate groups to the surface of the chitosan fibers, blocking the amino groups which helps prevent water adsorption and swelling of the fibers [47]. However, after a few seconds, there may be rearrangement of the chitosan – elastin – tBOC molecules with the elastin migrating to the surface of the fibers due hydrophilic amino acids (lysine, valine, and proline) that make up elastin’s structure [48]. We observed that this effect would take place immediately upon water exposure and would complete within 30 s. Therefore, to account for this change in material behavior over time, it was necessary to evaluate the water contact angle at two timepoints: (1) initial water contact t = 0 s and (2) after allowing water droplet to settle t = 30 s. 

Degradation results also suggest that elastin incorporation reduces the hydrophobicity of the membrane, as CE and CEMg groups degraded at a quicker rate than the C and CMg counterparts. The inclusion of MgP in the CEMg, and the increased fiber diameter, likely further disrupted fiber chain packing as compared to CE causing the slightly increased degradation. This effect can be taken advantage of by modulating elastin concentration to control membrane degradation rates which can be utilized for controlled drug-release. 

Elastin and MgP incorporation did affect the mechanical properties compared to the unmodified chitosan membranes. The differences seen in the tensile testing results may be attributed to the varying fiber diameters, polymer densities, and interactions among active components. Immediately after spinning, there are varying thicknesses of all materials due to the variations cause by the electrospinning process and its sensitivity to parameters like humidity and ambient temperature, which are difficult to precisely control. These thicknesses can reach upwards of 0.5 cm when untreated. All materials following tBOC treatment have a thickness of approximately 0.13 mm due to a flattening step that must be taken during the drying phase of the treatment. Additionally, the inclusion of magnesium and its ionic interaction with chitosan may be altering the effect that elastin provides to the material, as seen in the moduli and max elongation. We believe that the decrease in max elongation for CE, CMg, and CEMg is caused by the increased polymer density of the materials as compared to C. The lack of elongation, however, is expected when considering the increases in strength and moduli as a result of elastin and/or MgP incorporation. Additionally, it is likely that the TFA solvent is harming the elastin structure, preventing it from crosslinking, which can explain the absence of elasticity, characteristic of elastin in vivo. 

It is possible that we could further improve the mechanical properties of the CE and CEMg membranes by introducing crosslinking. Within the body, elastin relies on crosslinking to function, and researchers have begun to implement this in some tissue engineering applications [30,31,49]. The moduli and UTS of the membrane groups are similar to those found in other chitosan studies [50,51]. It may be possible to further improve the mechanical properties by incorporating fiber alignment or crosslinking to the membrane groups [50]. 

The MgP morphology was shown to be crystalline and elongated which may explain the large diameter measurement as it accounted for some particles along the long axis. However, it is unlikely that this morphology influenced the performance of the membranes as the crystalline structure of the MgP particles was likely lost due to the reaction and dissolution in TFA acid in the electrospinning solution, as seen by the lack of peaks in the XRD analysis of post-treated membranes The electrospinning protocol could be modified with a different solvent, like 1,1,1,3,3,3-Hexafluoro-2-propanol (HFIP), that might be more tolerable for MgP and retaining its structure. However, ash content combustion analysis indicated that the presence of MgP is near the expected value (~5 wt%) based on theoretical calculations. This suggest that little MgP is lost during the electrospinning, and post-treatment processes.

MgP release of 0.25 mM within the first 24 hours is comparable to other wound healing biomaterials that claim a “rapid” release of similar bioactive additives including silver and silica, however most materials aim for a controlled, stimulus-responsive release which is ideal for long term healing [52,53,54]. The concentration of MgP loaded and released from membrane was chosen as baseline to determine the feasibility of incorporation and ability to be retained within fibers through the electrospinning fabrication and treatment processes.

Cytotoxicity of MgP was only found to be at 1 mg and 10 mg MgP/mL medium, which suggests that NIH3T3 cells can tolerate exposure to particles at high concentrations. Within 3 days the 1mg MgP/mL medium group, which appeared cytotoxic on day 1, saw a large increase in cell count, comparable to other non-toxic groups. If the TAF solvent.

Overall, elastin incorporation improved the biocompatibility of ESCMs with fibroblasts. CE and CEMg membranes, exhibited the best proliferation rates highlighting the beneficial effects of elastin in supporting cell attachment and growth. This decreased hydrophobicity may be attractive to the fibroblasts, whereas extremely hydrophobic materials (like C and CMg) are not [55,56]. The immunofluorescence images of CE and CEMg showed differences in cell morphology, notably increased cell spreading, that may be attributed to elastin [57,58]. It should be noted that although the CMg membrane had the lowest cytocompatibility, the presence of MgP did not deter the CEMg membranes’ cytocompatibility.

There is currently very little research available on electrospun chitosan-elastin or CEMg biomaterials. Though there are many studies involving chitosan alone, but elastin has been noted to be difficult to mix with polymers (specifically synthetic) due to its insoluble structure and hence its popularity and usage as a biomaterial is reduced [59]. However, the increased bioactivity and mechanical properties that elastin provides are still sought after, which has led researchers to instead use elastin hydrolysates and elastin-like-polypeptides instead of natural elastin [59,60]. 

Further work on this material would include dosage loading and release of MgP and further post-treatment processes to allow for an extended release of MgP would be necessary before clinical use. Additional cell studies including other skin cell types like endothelial cells and keratinocytes are necessary to further test the material’s potential to support angiogenesis and full-thickness skin healing. Finally, in vivo studies, like a rat-skin or rabbit-ear excision/burn wound model, would be relevant to test the animals’ systemic response and antibacterial properties of the material. 

## 4. Materials and Methods

### 4.1. Membrane Fabrication

ESCMs were fabricated as previously described [28,47,61]. Briefly, a 5.5 (*w/v*)% Chitosan (Primex, Siglufjörður, Iceland) (71% DDA, MW = 311.5 kDa) was dissolved in trifluoroacetic acid/dichloromethane (TFA/DCM) (7:3) solution and mixed overnight on shaker at room temperature. To make chitosan-elastin (CE) electrospun membranes, 4 (*w/v*)% soluble, bovine-neck elastin “ES-12” (Elastin Products Company, Inc. Owensville, MO, USA) added to the chitosan-TFA/DCM solution the following day, just before spinning. Chitosan-MgP (CMg) and chitosan-elastin-MgP (CEMg) membranes were made by adding 0.5 (*w/v*)% MgP powder directly to C-TFA/DCM or CE-TFA/DCM solutions, respectively. After addition of elastin and/or MgP, solutions were vortexed for 1 minute and allowed to mix an additional 10 mins on shaker before transferring solutions to 10 mL syringe. These solutions were electrospun at 27 kV (Gamma High Voltage Research, Ormond Beach, FL, USA) in a custom plexiglass box vented to a fume hood used to make standard ESCM (C). After spinning, all membranes underwent a triethylamine/di-tert-butyl dicarbonate treatment to remove residual TFA salts and improve retention of fiber morphology in aqueous environments [29,47]. Briefly, membranes were soaked and mixed in a 10 (*v/v*)% triethylamine/acetone solution for 24 h. After rinsing with acetone three times, for 2 h each rinse, the membranes were transferred to a 0.1 g/mL di-tert-butyl decarbonate/tetrahydrofuran solution and mixed for 48 h. Membranes were removed and allowed to dry between nylon mesh sheets.

### 4.2. Magnesium-Phopshpate Particle (MgP) Synthesis

MgP were fabricated using a protocol established by Zhou et al. [37]. Microwave-assisted spontaneous precipitation was used to create MgP from a supersaturated biomimetic fluid. Briefly, sodium bicarbonate, magnesium chloride hexahydrate, and monopotassium phosphate are mixed in deionized water, then microwaved for 5 min, and allowed to cool to room temperature. This solution is poured into dialysis tubing (Spectra/Por® dialysis membrane; MWCO: 3500) and placed in 1 L of DI water, with water changes daily for three days. Dialyzed contents are then removed from tubing, allowed to freeze overnight, and then lyophilized for 48 h. 

### 4.3. Characterization 

#### 4.3.1. Scanning Electron Microscopy (SEM) and Energy-Dispersive X-ray Spectroscopy (EDS) Analysis

Electrospun fibers’ surface morphology among membrane groups was analyzed before and after tBOC treatment by SEM (NOVA NanoSEM 650, FEI Company, Hillsboro, OR, USA) and EDS analysis (Oxford Instruments NanoAnalysis, USA). Membranes were attached to a SEM stub with double-sided, conductive tape and sputter-coated with 5 nm gold-palladium.

Images were taken between 500× and 5000× magnification. Images of three sample locations per membrane and 20 fiber measurements per representative image of the membrane were taken to determine the uniformity of fiber morphology and average fiber diameter. Fiber analysis was conducted using ImageJ-Fiji analysis software, an open source plugin package for ImageJ. software (Fiji: version 2.9.0, developer(s): J. Schindelin et al., https://fiji.sc/; Image-J: National Institutes of Health, Madison, WI, USA).

EDS data was collected to determine TFA salt removal from spun membranes before and after the tBOC treatment via the F-peak. EDS was also used to determine MgP incorporation via the detection of the magnesium and phosphorus peaks. Spectra were analyzed via Aztec 3.0 software (Oxford Instruments, Abington, UK). 

#### 4.3.2. FTIR Analysis 

To verify the removal of TFA salts, attachment of hydrophobic group, and confirm elastin incorporation, Fourier Transform Infrared Spectroscopy (FTIR) (Frontier Universal Attenuated Total Reflection (ATR-IR) system with a diamond crystal, Perkin Elmer, Waltham, MA, USA). TFA-related peaks were investigated at 720, 802, and 837 cm^−1^. Hydrophobic treatment-related peaks would be found at 1370, 1529, 1688, and 2980 cm^−1^. Furthermore, elastin-related peaks were located at 1535 and 1655 cm^−1^.

#### 4.3.3. X-Ray Diffraction (XRD) Crystallography Analysis

XRD analysis was conducted on MgP alone and post-tBOC treatment electrospun membranes to characterize the particles’ effects on membrane crystallinity. XRD for all samples were collected using Bruker D8 Advance diffractometer (Bruker, Germany) outfitted with Cu Kα (∼0.154056 nm) radiation operated at a voltage of 40 kV and a current set at 40 mA with the 2θ range from 10° to 70° and a step size 0.05°. 

#### 4.3.4. Water Contact Angle Analysis 

Water contact angle measurements (n = 3) (VCA Optima Measurements Machine, AST Products, Billerica, MA, USA) were conducted on membrane groups to evaluate elastin and MgP incorporation’s effect on the hydrophobic treatment. A 5 μL droplet of water was extruded from the VCA automated syringe, and the material was raised up to the droplet to make contact, then lowered to allow droplet to settle on material. To observe the change in material behavior upon water exposure, water contact angle was measured upon initial water contact (t = 0 s) and after allowing the same droplet to settle for 30 s (t = 30 s). High resolution images were taken with the VCA camera. These images were used to measure the water contact angle by calculating the angle between the surface of the membrane and a line tangent to the droplet edge. 

#### 4.3.5. Immunofluorescence Staining for Elastin Incorporation

Single samples from membranes were qualitatively analyzed using indirect immunofluorescence staining to verify elastin incorporation. Anti-elastin antibody (ab21610, Abcam plc., Cambridge, U.K.) was used as the primary binder to elastin and conjugated with Alexa Fluor 488. Membranes were imaged using a Nikon Ti-E A1rSi confocal laser scanning microscope (Nikon Instruments Inc., Melville, NY, USA).

#### 4.3.6. MgP Size and Zeta Potential Analysis

MgP morphology was assessed using SEM imaging (NOVA NanoSEM 650, FEI Company, Hillsboro, OR, USA). A sample of MgP was spread across a SEM stub with double-sided, conductive tape and sputter-coated with 5 nm gold-palladium before imaging. 

The hydrodynamic particle size distribution and zeta potential of samples dispersed in deionized water were measured by dynamic light scattering (Delsa Nano C, Beckman Coulter, Brea, CA, USA), and the median size (number basis) was determined. Then, the average particle size and zeta potential were measured by using a flow cell with a scattering angle of 15°. The zeta potentials of MgP were determined from their electrophoretic mobilities according to Smoluchowski’s equation [62]. Measurement was taken as an average of three distinct measurements of the observed zeta potential and particle size.

#### 4.3.7. Combustion Analysis

Combustion analyses were used to assess incorporation of MgP incorporation into membranes. As the membranes are combusted, water and flammable organic components (chitosan and elastin) are removed, and remaining ash residue would be largely due to any incorporated MgP mineral. Post-treated C, CE, CMg, and CEMg membranes were placed in ceramic crucibles and allowed to combust in an oven at 550 °C for 3 h. Remaining mass was compared to crucible mass alone to calculate residual ash content (%). This test was used to confirm the differences in inorganic content between MgP incorporated (CMg and CEMg) and non-incorporated membranes (C and CE).

#### 4.3.8. Tensile Testing

Changes in ESCM tensile properties due to elastin and Mg NP incorporation (n = 4/membrane type) were evaluated using an InstronTM tensile testing system (Model 4456, Instron Mechanical Testing Systems, Norwood, MA, USA). Membranes were punched into dog-bone shape specimens with 20 mm gauge lengths and tested in tension using a 50 N load cell and an extension rate of 1 mm/min. Data were used to analyze the total percent elongation, ultimate tensile strength (UTS), and Young’s modulus of the different membrane groups.

### 4.4. In Vitro Analysis

#### 4.4.1. In Vitro Magnesium Release 

Discs were punched (diameter = 1 cm) out of CMg and CEMg membranes (n = 3/membrane type) and placed in 400 µL of PBS in 48-well plates to examine Mg ion release. Membranes were incubated at 37 °C, and supernatants were removed entirely and replaced at days 1, 3, 5, and 7. Eluates were evaluated for Mg ion release using the QuantiChrom™ Magnesium Assay Kit (BioAssay Systems, Hayward, CA, USA).

#### 4.4.2. In Vitro Degradation Study

A four-week degradation study (n = 4) was conducted using simulated body fluid (300 µg/mL lysozyme type VI [MP Biomedicals, Illkirch, France] in 1× PBS) to elucidate degradation profiles. Membranes were punched out into 1 cm discs and had original mass recorded. Samples were placed in 400 µL of simulated body fluid in 48-well plates and had solution changed every three days. Membranes were removed at weeks 1, 2, 3, and 4 and dried in a desiccator for 48 h before recording the remaining mass. Degradation progress was assessed by comparing membrane mass to original, undegraded membrane mass. 

#### 4.4.3. In Vitro MgP Cytotoxicity 

Cytotoxicity of the MgP was evaluated in vitro using NIH3T3 fibroblasts (ATCC#CRL-1658™). Two 96-well plates were seeded with 10,000 cells/well and allowed to adhere overnight in standard complete culture medium (α-MEM with 10% FBS, 500 I.U./mL penicillin, 500 µg/mL streptomycin, and 25 µg/mL amphotericin-B). A serial dilution of MgP in complete medium was made ranging from a concentration of 10 mg/mL to 0.1 ng/ml MgP/medium. This was used to replace the normal medium after 24 h post-seeding (n = 6/dilution). Cytotoxicity was assessed using CellTiter-AQ® assay (Promega, Madison, WI, USA) at 24 and 72 h post-exposure to MgP medium. 

#### 4.4.4. In Vitro Cytocompatibility of Membranes with NIH3T3 Fibroblasts

NIH 3T3 fibroblasts (ATCC#CRL-1658™) were used to evaluate the cytocompatibility of the membranes. Membranes (n = 5/group) were cut into 1 cm discs to fit the wells of a 48-well plate. These discs were sterilized via ethylene oxide gas sterilization. The culture medium consisted of α-MEM with 10% FBS, 500 I.U./mL penicillin, 500 µg/mL streptomycin, and 25 µg/mL amphotericin-B at 37 °C. Cells were seeded at 3 × 10^4/membrane in 48-well plates and evaluated for growth using the CellTiter-Glo® Luminescent Assay (Promega, Madison, WI, USA) at days 1, 3, and 5. A sample from each group was analyzed with fluorescent viability staining (LIVE/DEAD™ Stain, Invitrogen, Waltham, MA, USA) and imaged on a fluorescent microscope. 

### 4.5. Statistical Analysis

All statistical analysis was done using the ‘Real Statistics’ plugin for Microsoft Excel. Fiber diameter, tensile testing, water contact angle, and MgP cytotoxicity results were analyzed using one-factor ANOVA with Tukey HSD post hoc analysis (α = 0.05). Analysis of fiber diameter of pre- and post-treated membranes was done using a paired *t*-test (α = 0.05). Magnesium release, degradation, and NIH3T3 cytocompatibility results were analyzed using a two-factor ANOVA with Tukey HSD post hoc analysis for interactions between membrane groups and time (α = 0.05). 

## 5. Conclusions

This work shows the successful incorporation of both elastin and MgP, separately and together, into ESCMs. Results show that elastin incorporation significantly decreases the material’s hydrophobicity following tBOC treatment. The decreased hydrophobicity led to faster degradation rates and improved NIH3T3 fibroblast compatibility as compared to the elastin-free membranes. MgP were able to be loaded directly into the electrospinning solution and were retained throughout the fabrication and treatment processes, though possibly in an alternate form due to interactions with TFA acid in electrospinning solvent. Dual incorporation of elastin and MgP likely interrupted polymer chain packing which led to differences in mechanical properties, namely modulus and elongation. Though some fine-tuning may be necessary regarding MgP dosage/release and elastin crosslinking, this work identifies chitosan-elastin copolymer membranes loaded with MgP as a potential platform for future skin tissue engineering applications. 

## Figures and Tables

**Table 1:** Average zeta potential (mV) and particle size of MgP (*n* = 3).

Zeta Potential (mV)	Diameter (nm)
−11.8 ± 0.11	1660 ± 140

**Table 2:** Ash content combustion analysis of C, CE, CMg, and CEMg membranes.

Membrane Type	Ash Content (%)
C	3.64
CE	2.95
CMg	6.92
CEMg	8.86
